# Limitations of sequence dissimilarity as a predictor of prokaryotic lineage

**DOI:** 10.1098/rsob.240302

**Published:** 2025-03-19

**Authors:** Alvar A. Lavin, Juan Rivas-Santisteban

**Affiliations:** ^1^ Department of Systems Biology, Centro Nacional de Biotecnología, Madrid, Spain; ^2^ Department of Biology and Biochemistry, University of Bath Milner Centre for Evolution, Bath, UK

**Keywords:** sequence dissimilarity, molecular clock, prokaryotic evolution, phylogenetic time, gene polymorphism, molecular evolution

## Abstract

The molecular clock rests upon the assumption that the observed changes among sequences capture the differentiation of lineages, or kinship, as dissimilarity increases with time. Although it has been questioned over the years, this paradigmatic principle continues to underlie the idea that the polymorphic space of a gene is so vast that it is unattainable in evolutionary time. Thus, the molecular clock has been used to obtain taxonomic annotations, proving to be very effective at delivering testable results. In this article, however, we ask how often this assumption leads to inaccuracies when inferring the lineage of prokaryotic genes. Thus, we open an interesting discussion by simulating, in realistic scenarios, the critical times in which specific 5S rRNA sequences of two distant lineages are exhausting the polymorphic space. We contend that certain genes in one lineage will become increasingly similar to those in another over time, as the space for new variants is finite, mimicking phylogenetic features by convergence or by chance, without implying true kinship.

**Figure:**
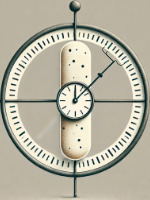

## 1. Introduction

The distinctiveness of the inheritable traits among the species is tied to genetic change. Classically, neo-Darwinists explained the variability of amino acid sequences in different lineages by events of positive selection over time [1,2]. For example, albumin proteins from close species retained the higher identity among sequences and structures, but the proteins from distant species were dissimilar [3]. Zuckerkandl & Pauling introduced the term 'molecular clock' to refer explicitly to the rate of occurrence of these amino acid substitutions between lineages, which they observed to be fairly linear with time [4]. Later on, the neutralists succeeded in establishing the statistical foundations of the neutral theory and relate it to the molecular clock, explaining the rate of appearance of polymorphisms beyond Darwinian evolution [5–7]. They argued that selective forces acting on gene evolution were the cause of the deviations in the constancy of the rate of the clock (accumulation of variants per unit time), since the rate would be constant in pure neutral evolution, where the extent of polymorphism, or θ, is contingent on the product of the effective population size and the mutation rate ($N_{e}\times \mu$). These explanations satisfied many of the observations in the animal genes studied [8]. At the same time, different statistical models of phylogenetic inference were proposed, which operate calculating arbitrary distances from changes in a set of nucleotide sequences [9,10]. So the field had both the tools and the theoretical corpus to interpret modern genomic data, hence the flourishing of phylogenetics. Though legitimate critiques of the molecular clock succeeded over the decades [11–15], the notion that sequence similarity of a gene can reliably indicate kinship has remained largely accepted, at first as a problem-solving model [16], and over time, as a foundational paradigm [17–20].

**Figure 1.**
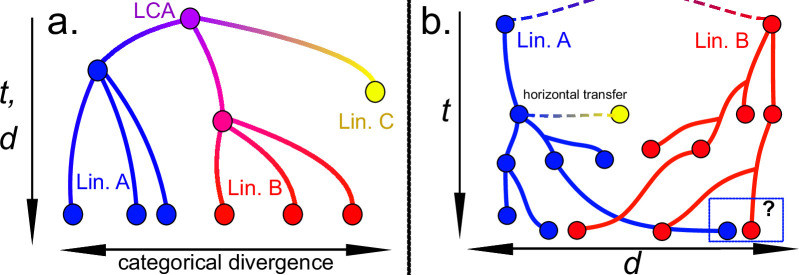
(a) Representation of the molecular clock assumption in which lineages (blue, red and yellow) are generated by accumulation of variation at a constant rate, μ, so t (time) is the same dimension as, or is deduced from, d (dissimilarity between sequences). Every point represents a specific gene sequence in a given time. We usually work with this assumption to infer phylogenetic time from current sequence variants, making taxonomical categories in the process. (b) t≠d. In this article, we argue that the probability of finding a sequence identical to another from a distant lineage must be non-zero—in fact, it should increase over certain critical time. Therefore, inferring time from divergence data would be prone to error (question mark at right bottom). In this work, we explore how to calculate the value of this probability. In addition, horizontal transfer (yellow) may be a diluent of the lineage signal.

Apart from this overdispersion of the clock, however, two other major issues of the molecular clock model to infer phylogenetic time should be indicated, especially among prokaryotes: (i) it neglects the effect of horizontal gene transfer (HGT) on molecular evolution; and (ii) it assumes that sequence divergence between two lineages will keep increasing linearly with time (figure 1a), even if we know that the phylogenetic signal saturates [21].

Regarding (i), we can exemplify how new evidence contradicts some of the initial claims. In 1975, Woese *et al*. showed that some sites of the 16S rRNA gene were fairly conserved among prokaryotic lineages [22]. Later, in 1980, Fox *et al*. raised some doubts on how well the observed differences among 16S rRNA gene sequences recalled kinship, although they acknowledged that dissimilarity coefficients can be used as a proxy of ‘their true phylogenetic relationships’ [23]. However, we are now aware that this gene can be inherited horizontally from different species, regardless of whether they belong to close [24,25] or to extremely distant [26,27] lineages. Moreover, prokaryotic lineages are not bounded to a genome size or whole gene sets. Instead, species often only share a succint ‘core’ genome [28], with significant portions of their genetic repertoire subject to dynamic flux via HGT [29]. This has more to do with a poor definition of bacterial species. Nevertheless, if issue (ii) were true—that divergence always increase with time—we could still distinguish the true lineage of the gene variants, irrespective of the bacterial species in which they are implemented. For that reason, let us focus now on issue (ii).

To better understand it, we will propose an entertaining exercise. Let us think of two genes (*gene X* and *gene Y*) arising at a given moment in the history of evolution on Earth. These two genes perform the same function in bacteria of two different species, which remain independent from each other (*lineage A* and *lineage B*, respectively). These genes are similar in structure and function, but comparatively dissimilar in sequence. We will suppose that the expression of each of these genes involve an increase in fitness to such a degree that they become fixed in their respective populations rapidly. These genes will accumulate neutral and adaptive changes over time. An interesting question to ask here is: how likely is it that the ‘progeny’ of *gene X* from *lineage A* will resemble the original *gene Y* from *lineage B*, and vice versa, given enough time? In the next sections, we will get a sense of the odds that some of the produced sequences revisit changes and increase in similarity (figure 1b), mimicking the other lineage by chance or by convergence, which would result in a poor linear fit between time and sequence dissimilarity or uniqueness.

## 2. Blind permutation of semantides

Assuming the absence of selection among semantides (informational polymers [4]), the probability of finding an identical sequence by blind permutation is given by


(2.1) $$P(L,k)={\left(\frac{1}{k}\right)}^{L}$$

where L is the length of the semantide and k is the number of unique monomers. For example, known semantides have *k* values of 4 (deoxy-/ribo-nucleotides) for nucleic acids, or *primary* and *secondary* semantides, and 21/22 (proteinogenic amino acids) for proteins, or *tertiary* semantides [30]. In contrast, *L* values are highly variable (few to thousands).

We will observe that the longer the length of the semantides, the greater the influence of the number of unique monomers on decreasing the odds. This makes it increasingly difficult to find an identical sequence by combinatorics, although still possible, as the length approaches that of the smallest protein genes. However, biological semantides are governed by explicit rules, involving ‘memory’ and ecological constraints on the exploration of the sequence space. Therefore, these probabilities need to be calculated integrally under evolutionary assumptions and realistic scenarios. This will be addressed in the following section.

## 3. Evolution of gene divergence in a finite polymorphic space

The neutral model of molecular evolution set the theoretical upper limit of polymorphism on populations of prokaryotic sequences as follows [8,31]:


(3.1) $$\theta \times n=\mu \times N\times 2^{n},$$

where θ is the number of polymorphisms of a gene, μ the accumulation of variation per site per generation, N the number of initial replicants (*gene copies* × *prokaryotic cells*) and n the number of generations. However, an assumption implicit in equation 2—and mandatory in the molecular clock—is that polymorphic variants are virtually infinite, whereas they are actually bounded by $k^{L}$ (equation 1). Although very high, the upper limit for polymorphic variants of a 120 bp gene length is not infinite ($4^{120}$). Even if the initial number of replicants (N) is considered to be 1, and the mutation rate one of the best known, yet slower, for bacteria [32,33]


$$4^{120}=2.54\times 10^{-10}\times 1\times 2^{n};$$

n=271.87.

Only 272 generations—equivalent to approximately 4 days in *Escherichia coli* [34]—would be needed to obtain all possible polymorphic variants of a 120 bp gene length, if generation time is maintained. Nevertheless, this assumes unlimited growth. One way to avoid this otherwise unrealistic assumption is to approximate the evolutionary population size or $N_{e}$, instead of $N\times 2^{n}$. A conservative result for haploids would be


$$4^{120}=2.54\times 10^{-10}\times 2\times 10^{8}\;\times n;$$

$$n=3.48\times 10^{73}$$

**Figure 2.**
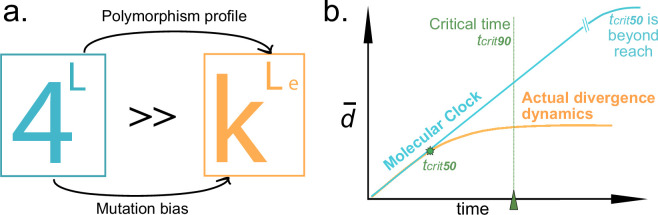
(a) Representation of the polymorphic space for gene of length Lbp, which is far smaller (orange) than it appears at first hand (light blue). Due to first purifying selection that constrains the variability of some sites contingent to variability in others, sometimes quite strictly, which drastically lowers the exponent elevating 4; and second to mutational biases, where the overwhelming difference between probabilities of transitions and transversions per site makes the actual base of the sequence space closer to 1 than to 4. (b) Time does not linearly correlate with divergence; rather, after a certain period, average sequence dissimilarity remains constant. $t_{crit_{50}}$ is reached within evolutionary time. In light blue (molecular clock), the polymorphic space is assumed so big that it is considered infinite, or alternatively, the time it takes to exit the linear range ($t_{crit_{50}}$) is far superior to any realistic evolutionary time. In orange, we argue that it is not that big and that could be exhausted within prokaryotic evolutionary time.

Taking approximately $1.32\times 10^{69}$ years to *E. coli* to achieve all the polymorphic variants for the given gene length. This number may be interpreted as infinite, far exceeding the age of the universe ($\approx 1.38\times 10^{10}$ years). If this were truly the case, the probability of mistaken kinship would be so low that the molecular clock becomes a very appropriate model. However, this conclusion is led by substantial inaccuracies. First, a semantic one, as maintenance of function is required to be considered the same gene. Otherwise, we would be calculating the potential polymorphism of all genes of the same length—not of a gene. Second, $N_{e}$ is governed by mutation rates and niche contingencies in prokaryotes, and therefore, its value varies across time and space [35–37] and within the elements of a genome [38]. Third, the weakly deleterious models consider species with big $N_{e}$—like most prokaryotes—as evolving with high selection and low drift [39]. Therefore, the actual number of gene polymorphisms is unlikely to be explored, as many would be deleterious. This is to say that the exponent that defines the polymorphic space for any natural gene is actually far smaller than its raw length, L, would indicate because each site along the gene has a limited amount of changes allowed by purifying selection, not only in and out of itself, but contingent to the changes on all other sites. Thus, in most genes, this value of ‘effective’ length ($L_{e}$) will be one arbitrarily lower than actual gene length, exponentially constraining the space. At last, the mathematical base of the space is likewise virtually smaller than 4, owing to the fact that profound asymmetry imparted by mutational biases (transitions over transversions [40]) in practice reduces the number of available alternatives for one base pair from three to a number closer to one (figure 2a). Under the assumption of a molecular clock, the linear range of the divergence can be presumed to describe the dynamics at any point because the critical time it would take to achieve saturation in the oversized naive model of the polymorphic space (blue in figure 2a) can be calculated as several ages of the universe. To the contrary, with the actual size of the polymorphic space (orange in figure 2a), this saturation is reached within feasible evolutionary times (figure 2b). This interpretation is in agreement with Dryden *et al*. [41].

Consequently, while the observed polymorphic diversity of a gene might appear astronomical, the number of generations needed to exhaust it in a real-world scenario should be substantially lower. So if the potential for polymorphic diversity, although grueling to drain, is not infinite due to selection and properties of the semantide (evolving length and number of distinct possible monomers per site), then specific sequences will be revisited to an array of degrees without precluding lineage, potentially debilitating phylostratigraphic approaches to infer gene age, for example [42,43].

## 4. The critical time $t_{crit}$ versus the molecular clock

The concept of the critical time is of key importance to understand this deviation from a pure molecular clock. Its fundamental assumption only becomes true as time approaches infinity, because only then does the relationship between time and kinship become perfectly linear. For finite time, there is always a degree of curvature. That this curvature should be negligible owing to gargantuan sequence spaces is the actual serious proposition underlying the practical value of molecular clock assumptions, as we explained in the previous section.

We have delimited two critical points that we consider of note for their prospective applicability, which we call the 50% critical time or $t_{crit50}$ and the 90% critical time or $t_{crit90}$. These times could provide microbiologists with two practical heuristics when managing phylogenetic reconstructions. $t_{crit50}$ represents the point in time at which divergence with respect to an ancestor reaches 50% of its maximum steady state value allowed by the available polymorphic space and ecological constraints of the host. This point corresponds roughly with the halfway exploration of the space and with the midpoint of the collapse of correlative strength between sequence dissimilarity and time. Trees reconstructed to feature sequences that diverged past $t_{crit50}$ should be sufficiently warped to warrant demerit as models, but still retain genuine information about phylogeny. The second point or $t_{crit90}$ corresponds to the time at which sequence dissimilarity with respect to the reference ancestor has reached 90% of the steady state value. Past this point, divergence becomes greatly invariant to time. Phylogenetic reconstructions lumping sequences past $t_{crit90}$ with sequences before this time may fail remarkably at placing the former in their rightful context. Past $t_{crit90}$, divergence settles onto saturation dynamics, and its approach to the asymptote past the uppermost percentile gets effectively clouded by mutational noise.

It is worth noting that a similar concept was pointed out earlier in the literature where $d_{n}/d_{s}$ comparisons between bacteria were found time-dependent [44]. The critical time therefore provides a way of assessing the reliability of phylogenetic reconstructions at first hand: if the common ancestor of an array of sequences lies possibly beyond estimates of $t_{crit50}$, a phylogenetic reconstruction based on arbitrary distances will demonstrably feature artefacts; if it lies possibly beyond $t_{crit90}$, the clock would be seriously violated. However, we first must perform realistic calculations of critical times for particular genes to know if they are attainable in evolutionary timescales.

## 5. Simulation of a real scenario: the 5S rRNA gene

To delimit the implications of this new perspective, we set out to simulate the evolution of a 120 bp gene with the above assumptions. We selected the 5S ribosomal RNA gene because it is well represented in the global phylogeny. In addition, its length, function and the secondary and tertiary structures remain widely conserved [45], although the sequences exhibit considerable variability [46]. We conducted evolutionary simulations incorporating key ecological parameters—such as mutation, extinction and duplication rates [32,33]—that influence the rate at which the 5S rRNA polymorphic space is explored. We did not consider indels which, in turn, are infrequent in 5S rRNA genes [45], nor HGT. These simulations focused on two distant prokaryotes: *Pseudomonas stutzeri* (bacterium) and *Halorubrum distributum* (archaeon). We found the critical times $t_{crit_{50}}$ for the case of their 5S genes to be as low as 9 Myr (electronic supplementary material, figure S1), a remarkably small span in geologic terms. After these critical times, the population of wild sequences had explored 50% of the sequence space as defined by the length, polymorphism profile (directly taken from the reference 5S sequences; electronic supplementary material S1) and mutational biases [40] along the sequences. The divergence with respect to any ancestor sequence thus peaked at around 0.315 for *P. stutzeri* and 0.296 for *H. distributum*, and the extant populations of sequences had standard deviations of 0.037 and 0.034. In line with the point showcased in the previous section, these results suggest that $t_{crit_{50}}$ for *P. stutzeri* may be as low as 29 Myr, and even shorter for *H. distributum* at 9 Myr. Even the $t_{crit_{90}}$ is reached within evolutionary times rather meek for considerations involving prokaryote evolution, with *P. stutzeri* having the longer one at 152 Myr.

**Figure 3.**
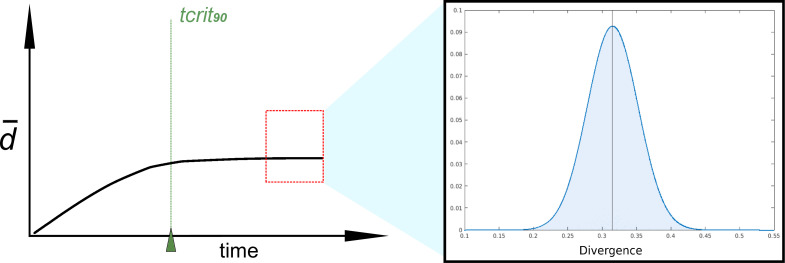
Steady state distribution of divergences after $t_{crit_{90}}$. Past $t_{crit_{90}}$ the distribution practically does not change with time, thus providing ample room for distortions in phylogenetic reconstructions assuming a molecular clock. The divergence distribution showed corresponds to *Pseudomonas stutzeri* 5S rRNA simulated evolution ($>t_{crit_{90}}$). Real simulated curves are shown in the electronic supplementary material (figure S1).

To further illustrate the implications of these problems for our study, let us now imagine any hypothetical prokaryote that happened to actually diverge from *P. stutzeri* or *H. distributum* at a point deep in geologic time, and higher than the critical time for their 5S sequences. If we tried to find this point in time (i.e. by a molecular clock) to reconstruct the phylogeny of the reference organisms and the problem organism, it is easy to see how, from the spectrum of extant sequences of *P. stutzeri* or *H. distributum*, we could take any of a number of divergences that would return artefactual levels of relatedness, completely disconnect from the actual kinship shared by the two groups (figure 3). Building a tree with sequences from populations before $t_{crit_{50}}$ is probably legitimate, but introducing any number of sequences from populations outside the range would introduce distortions because kinship may be overestimated (electronic supplementary material, figure S2).

## 6. Concluding remarks and open questions

Although we discussed how polymorphic space can be exhausted in a realistic scenario within short evolutionary times, we did not consider the role of indels in gene evolution. These events are likely to modify the exponent $L_{e}$ of the evolving gene, making the task of distinguishing kinship easier. In top of that, larger genes may retain a significantly bigger polymorphic space. In those cases, the linear range until reaching $t_{crit_{50}}$ will become extensive and thus synonym with a molecular clock. Finally, calculating $t_{crit_{50}}$ for eukaryotes may yield limited results, given their typically smaller $N_{e}$ (and therefore a greater susceptibility to non-Darwinian evolution), their considerably longer generation times and the extensive genomic regulation (splicing). In general, all these effects will act extending the linear range in time.

Nevertheless, we contend that this work provides a foundational basis for ongoing discourse. A legitimate conclusion of polymorphic spaces being finite and attainable is that we may be incurring in phylogenetic distortions when comparing genes that diverged within a time range spanning from a few million to several hundred million years ago. The probability of sequence revisitation is therefore non-zero and is submaximal after the critical time $t_{crit_{90}}$. Assuming neutral variation on unrestricted sites, the linear correlation between time and kinship decreases from $t_{crit_{50}}$ onward and becomes almost null at $t_{crit_{90}}$, well within that from phylogenetic reconstructions.

Further investigation may answer some new open questions. We enumerate four important ones: (i) how our arguments may apply for other, larger genes; (ii) how differential $t_{crit}$ in the horizontally transferred materials may compound the situation; (iii) how the coalescence of lineages, particularly through endosymbiosis, might mask the parameters of the polymorphic space for a gene; and (iv) how this finite polymorphic space, which makes the gene sequences prone to convergence, may affect the notion of prokaryotic species.

## Data Availability

All reference and generated sequences and code is available on electronic supplementary materials. Supplementary material is available online [47].
